# Impact of preoperative atrial fibrillation on mortality and cardiovascular outcomes in patients undergoing double valve surgery

**DOI:** 10.1186/1749-8090-8-S1-O64

**Published:** 2013-09-11

**Authors:** M Urban, J Pirk, O Szarszoi, P Ivak, I Netuka

**Affiliations:** 1 Department of Cardiac Surgery, Institute for Clinical and Experimental Surgery, Prague, Czech Republic

## Background

The prognostic significance of atrial fibrillation (AF) on postoperative outcomes and survival of patients following concomitant aortic and mitral valve surgery remains unclear. The aim of his study was to assess the impact of the presence of the preoperative AF on the outcome.

## Methods

This was a retrospective study of 341 patients who underwent double valve replacement with either tissue (n=164) or mechanical (n=177) prostheses. The patients were divided into two groups according to preoperative rhythm status. Demographic, clinical and echocardiographic data were extracted from the hospital database and patient notes. Follow-up data were gathered through outpatient visits or mailed structured questionnaire. Data were analyzed for major adverse valve related complications and survival.

## Results

Follow-up was 93% complete and totaled 1398 patient-years. Although patients with preoperative AF were significantly older, presented with higher logistic EuroScore and larger left atria on echocardiographic examination, five year survival was comparable (83 ± 4% SR vs. 75 ± 4% AF, p = 0.194). Patients with preoperative AF who underwent replacement with tissue valves with MAZE had 68 ± 10% five year survival in comparison to 38 ± 11% without MAZE procedure (p=0.032). There was no difference in survival in patients who underwent replacement with mechanical prostheses with or without MAZE (85 ± 7% vs. 87 ± 3%, p=0.216). Freedom from major adverse valve related complications at five years was 81 ± 4% in SR and 73 ± 5% in AF group (p=0.189). Multivariate analysis identified older age, higher EuroScore, preoperative renal insufficiency and concomitant CABG as significant adverse predictors for overall survival.

## Conclusions

There was no difference in postoperative morbidity and mortality in patients with AF versus those in SR. In patients with preoperative AF who underwent valve replacement with tissue prostheses MAZE offers significant survival advantage.

